# Supplementary material for: Causal interplay between lactose intolerance and gut microbiota: a combined bidirectional Mendelian randomization and in vivo validation study
Source: Front Nutr. 2026 Jun 1;13:1803337. doi: 10.3389/fnut.2026.1803337 (PMC13265576; doi:10.3389/fnut.2026.1803337)
Supplement: Supplementary file 1 [file Data_Sheet_1.zip › supplementary materials/Forward/leave one out cross validation/ebi-a-GCST90027686.finngen_R12_E4_LACTONAS.pdf]

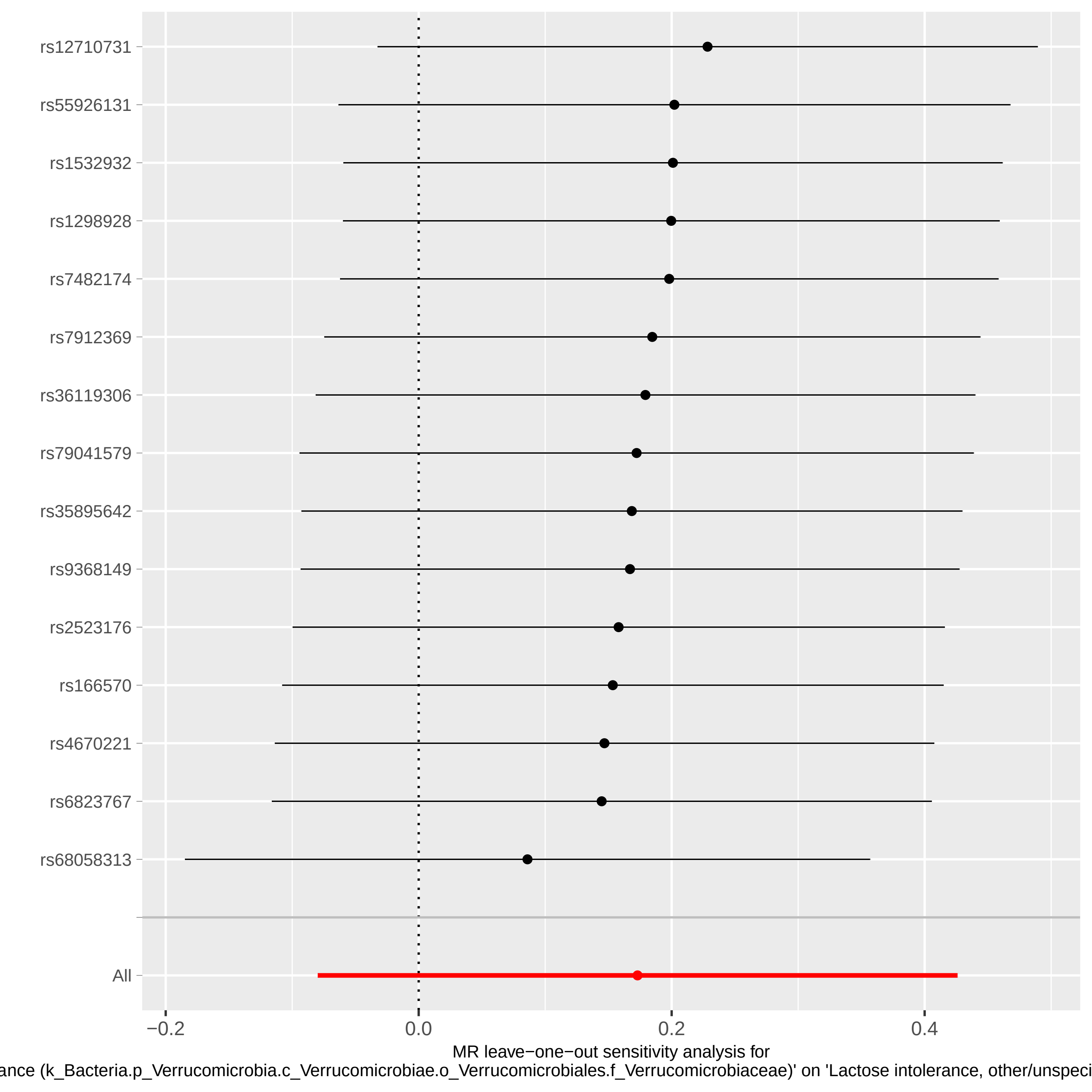

rs12710731
rs55926131
rs1532932
rs1298928
rs7482174
rs7912369
rs36119306
rs79041579
rs35895642
rs9368149
rs2523176
rs166570
rs4670221
rs6823767
rs68058313
All
−0.2
0.0
0.2
0.4
MR leave−one−out sensitivity analysis for
ance (k_Bacteria.p_Verrucomicrobia.c_Verrucomicrobiae.o_Verrucomicrobiales.f_Verrucomicrobiaceae)' on 'Lactose intolerance, other/unspeci
